# Number of Eating Occasions and Source of Foods and Drinks Among Young Children in the United States: NHANES, 2009–2014

**DOI:** 10.3390/nu11040897

**Published:** 2019-04-21

**Authors:** Chloe M. Barrera, Latetia V. Moore, Cria G. Perrine, Heather C. Hamner

**Affiliations:** Division of Nutrition, Physical Activity, and Obesity, National Center for Chronic Disease Prevention and Health Promotion, Centers for Disease Control and Prevention, 4770 Buford Hwy NE, Mailstop F-77 Atlanta, GA 30341, USA; ggi9@cdc.gov (L.V.M.); hgk3@cdc.gov (C.G.P.); hfc2@cdc.gov (H.C.H.)

**Keywords:** child, meal patterns, food source, National Health and Nutrition Examination Survey

## Abstract

An understanding of the source of children’s foods and drinks is needed to identify the best intervention points for programs and policies aimed at improving children’s diets. The mean number and type of eating occasions and the relative proportions of foods and drinks consumed from different sources were calculated among children aged 1–4 years (*n* = 2640) using data from the 2009–2014 National Health and Nutrition Examination Surveys. Children consumed 2.9 meals and 2.4 snacks each day. Among children who received anything from childcare, childcare provided 36.2% of their foods and drinks. The majority of foods and drinks came from stores for all children (53.2% among those receiving anything from childcare and 84.9% among those not). Among children receiving food from childcare, childcare is an important source of foods and drinks. Because most foods and drinks consumed by children come from stores, parents and caregivers may benefit from nutrition education to promote healthful choices when buying foods.

## 1. Introduction

Eating habits developed during infancy, early childhood, and adolescence may impact later eating patterns [1,2]. The diet consumed during early childhood may also influence children’s cognitive development, behavior, and growth [3,4]. Furthermore, a poor early diet has the potential to increase the chance of developing obesity later in life [5,6]. 

The 2015–2020 US Dietary Guidelines for Americans (DGA) describe what constitutes healthy eating patterns and the importance of a healthy and nutritionally adequate diet for the US population [7]. However, the DGA does not offer guidance for children younger than 2 years of age. The American Academy of Pediatrics (AAP) offers guidance on the number of meals and snacks in order to provide a constant supply of nutrients to fuel children’s activities; specifically, children 1 to 4 years of age should be given three main meals and two to three snacks spread throughout the day [8]. However, there is scant recent data on how many meals young children are consuming. One study reported that there is an increasing trend in the number of meals and snacks (from here on termed eating occasions) consumed in a day among representative samples of US children aged 2–18 years, from three per day in 1977 to five per day in 2006 [9].

In addition to the potential importance of the number and type of meal and snack offered, data show that where food is obtained is important. According to the US Department of Education’s National Household Education Survey, 41% of children aged 0–5 years are cared for weekly in nonrelative care arrangements (e.g., childcare centers, family care homes, prekindergarten classrooms, and head start programs) [10] and likely consume multiple meals in this setting. The number of children in childcare increases by age with 19% of children aged younger than 1 year, 32% of children aged 1–2 years, and 64% of children aged 3–5 years in nonrelative care [10]. Some childcare centers provide food for children, while others expect children to bring meals and snacks from home. Although childcare may be an important source of food for young children, few studies have researched the proportion of foods and drinks provided by this setting.

An understanding of the number of eating occasions and the proportion of foods and beverages provided to young children by childcare, as well as other sources, may support public health efforts to identify the best intervention points for programs and policies aimed at improving young children’s diets. To fill this research gap, this study describes the number of meals and snacks that children in the US consume throughout the day, as well as the source of the foods and drinks that make up those eating occasions. To our knowledge, this is the first nationally representative analysis describing these aspects of young children’s meal patterns.

## 2. Materials and Methods 

### 2.1. Data Source 

Data from the 2009–2010, 2011–2012, and 2013–2014 National Health and Nutrition Examination Survey (NHANES) were combined and used in this analysis. NHANES is conducted in 2-year cycles by the US Centers for Disease Control and Prevention’s National Center for Health Statistics. Each 2-year cycle obtains nationally representative data on the health and nutritional status of the noninstitutionalized civilian population in the United States and uses a complex, stratified, multistage probability design to select participants. Respondents participate in both a household questionnaire and a physical examination at the Mobile Examination Center (MEC). Details on the survey are described elsewhere [11]. The National Center for Health Statistics Research Ethics Review Board approved all NHANES protocols, and a proxy provided written, informed consent for all participants.

### 2.2. Analytic Sample

Among the three survey cycles, 2,966 children aged 1 to 4 years were interviewed (via a parent or guardian proxy) and completed a physical examination in the MEC. MEC unweighted response rates for this age group ranged from 74.6%–86.8% [12]. Children were excluded if they did not have complete and reliable 24-h dietary recall data (*n* = 326), as determined by the National Center for Health Statistics, for the first dietary recall interview. This resulted in a final analytic sample of 2640 participants. We limited the sample to children aged at least one year as participants under one may have been receiving a large portion of their meals in the form of breast milk or infant formula. 

### 2.3. Covariates 

Information on age, sex, race/Hispanic origin, and the ratio of household income to federal poverty threshold was collected during the in-home interview. Age was dichotomized into two age groups: (1) 1 to 2 years of age (including children aged 1 to less than 3 years) and (2) 3 to 4 years of age (including children aged 3 to less than 5 years). Race/Hispanic origin was based on answers to questions about race and Hispanic origin. For analyses stratified by race/Hispanic origin, we reported three categories: (1) non-Hispanic white, (2) non-Hispanic black, and (3) Hispanic. We do not present estimates for the other race/Hispanic origin category or for the non-Hispanic Asian origin category (included only in the 2011–2014 survey cycles) because of limited sample sizes. However, these data are included in the analyses. The ratio of household income to the federal poverty threshold was calculated for each NHANES cycle by using annually updated Department of Health and Human Services’ poverty guidelines [11]. The income to poverty ratio was dichotomized to income to poverty ratios more than 1.85 and income to poverty ratios less than or equal to 1.85 as this is the threshold used for the federal Special Supplemental Nutrition Program for Women, Infants, and Children (WIC) eligibility [13]. 

### 2.4. Dietary Assessment 

A 24-h dietary recall was collected at the MEC by trained interviewers using the automated multiple-pass methodology (AMPM); further dietary recall procedures are described elsewhere [14,15,16]. A proxy, typically the child’s parent, responded to the 24-h dietary recall for all participants. Detailed information was collected on each food and drink item consumed, including the name of the eating occasion, the clock time of consumption, and the source. 

A list of 20 different types of eating occasions was provided to the respondent for selection; however, eating occasion names were not defined for the respondent. We categorized these into five meal categories: (1) breakfast (breakfast, *desayuno*, and *almuerzo*); (2) lunch (lunch, *comida*, and brunch); (3) dinner (dinner, supper, and *cena*); (4) snack (snack, *merienda*, *entre comida*, *botana*, *bocadillo*, *tentempié*, extended consumption, drink, bebida, and infant feeding); and (5) other. Infant feeding most commonly included infant formula or breast milk. No participants listed other for their eating occasion; therefore, this category was not included in tables or further analysis. All foods and drinks recorded at the same clock time and reported eating occasion (e.g., breakfast) were considered one eating occasion. In line with previous studies, food or drink items recorded with start times longer than 15 min apart were considered different eating occasions [17]. Therefore, if a respondent reported an eating occasion of breakfast at 8:15 am, as well as 9:30 am, the respondent was considered to have had two breakfasts. 

During 2009–2010, participants were given 24 options for the source of each food or drink item; these were expanded to 26 options during 2011–2014. Specifically, “store” was further broken down to “store—convenience type” and “store—no additional info.” We collapsed the different source options into four categories: (1) store-bought foods (retail stores including grocery, supermarket, convenience, and store—no additional info); (2) restaurants (restaurants, street vendors, vending trucks, bar/tavern/lounge, and sports, recreation or entertainment facilities); (3) childcare (school and other institutional cafeterias, child/adult care center, and child/adult home care); and (4) other source (soup kitchens or food pantries, Meals On Wheels, other community food programs, residential dining facilities, vending machines, common snack tray, homegrown or gifted, mail-order purchase, and any other reported source). Eating occasions consisting of only water were excluded from the source of food and beverage analysis because “tap water” was given its own source variable different from that of other foods and beverages and, therefore, could not be categorized in the same way. 

### 2.5. Statistical Analysis 

Demographic characteristics were calculated separately for children aged 1 to 4 years. We calculated the mean number of eating occasions in a day, and weighted estimates are presented. To compare how many food and drink items came from each source (store, restaurant, childcare, or another source), the ratios of the means were calculated by dividing the sum of the number of food and drink items for each source by the sum of the number of food and drink items consumed by the population (excluding water) to estimate the relative percentages [18].

All statistical analyses were performed overall and separately for children aged 1 to 2 years and children aged 3 to 4 years and logistic regression was used to assess whether patterns varied by age. A *p*-value of less than 0.05 was considered statistically significant. SAS survey procedures were used to account for the complex survey design of NHANES (SAS Version 9.3, SAS Institute, Inc., Cary, NC, USA). A 6-year sample weight using day one dietary recall weights were used for weighting [19].

## 3. Results 

Table 1 illustrates the selected sociodemographic characteristics of the 2640 children. More than half (50.6%) of the children were aged 1 to 2 years. Approximately half of the children were male (51.0%); 51.2% were non-Hispanic white, 14.0% were non-Hispanic black, and 25.2% were Hispanic. Less than half (46.7%) had a ratio of family income to poverty threshold more than 1.85. 

Overall, children aged 1 to 4 years had 5.3 eating occasions each day (1.0 breakfast, 0.9 lunch, 1.0 dinner, and 2.4 snacks). Of the snacking occasions, 0.5 were a drink, and 0.1 were an infant feeding. Overall, 3- to 4-year-olds had about 8% fewer eating occasions than 1- to 2-year-olds (5.1, 5.6, respectively; *p* < 0.0001). Most of these differences are accounted for in drinks and infant feedings. 

Among children aged 1 to 4 years, 9.7% received food from childcare on a given day. Table 2 describes the source of all foods and drinks reportedly consumed in a 24-h recall by children aged 1 to 2 years and 3 to 4 years stratified by whether or not they had received any food from childcare. Stores were the primary source of foods and drinks regardless of age or whether or not food was received from childcare. Among children who received any food from childcare, store-bought foods comprised 53.2% of their daily intake whereas foods from childcare comprised 36.2% of their daily intake. Among children who received no food from childcare, store-bought foods comprised 84.9% of their daily intake. 

## 4. Discussion

This study provides insight into the eating patterns of children aged 1 to 4 years in the United States. We found that, on average, children are meeting eating occasion frequency recommendations from the AAP for children aged 1 to 4 to consume three meals and two to three snacks per day [8]. Most of the foods and drinks that children this age consume are store bought and about 10% receive food from childcare. 

There are programs in place to promote healthy nutrition among young children and to help children get regular meals both at home and away from home. WIC works to ensure that all children under age 5 who are at nutritional risk receive nutritious foods [20]. Children who are at nutritional risk include those who have nutrition-related medical conditions, dietary deficiencies that impair or endanger health, and conditions that predispose them to inadequate nutritional patterns or other nutrition-related medical concerns [20]. Another program, the Child and Adult Care Food Program (CACFP), helps to provide nutritious meals and snacks to childcare centers [21]. Among children who receive food from childcare, foods and drinks from childcare make up about 42% of the diet of 1- to 2-year-olds and about 34% of the diet of 3- to 4-year-olds. This suggests that many childcare centers are a significant source of food for the children in their care.

Given that our findings and other research among older children and adults have also found that the majority of food and drink items come from a store [22], resources designed to be used in stores may help parents and caregivers in their purchasing and preparing food for the entire family. Stores offer a unique setting to intervene, and point-of-purchase interventions have been implemented in grocery stores with success [23,24]. One such intervention implemented a “Healthy Kids Kiosk,” of which 58% of users reported that the kiosk encouraged them to buy healthier foods [23].

A third important source of foods and drinks for young children is restaurants (6.7% for children receiving food from childcare and 8.9% for children not receiving any food from childcare). A previous study found that children consume significantly more energy from fat and saturated fat when they eat at restaurants compared with any other location [25]. Helping decision makers opt for healthier choices when eating outside the home may also be helpful.

This study had limitations. Misclassification errors could have occurred as a result of incorrect categorizations of eating occasion and source variables, as well as differing definitions of what constitutes an eating occasion or source. In addition, dietary recall data are based on self-report and may be subject to reporting errors; this may be particularly true in the case of proxy recall.

Strengths of this study include that NHANES is a nationally representative data source and, therefore, our findings are generalizable across the United States. In addition, interviews were conducted in person by a trained interviewer and eating frequency estimates are based on 24-h dietary recall data. 

## 5. Conclusions

This study describes the mean number of eating occasions among young children in the United States and the source of young children’s foods and drinks. Overall, we found that children aged 1 to 4 years have about five eating occasions in a day, for which most foods and drinks come from store-bought foods. However, among the nearly 10% of children who received food from childcare, this source contributed to over a third of foods and drinks. Considering food source information when designing interventions aimed at ensuring young children have healthy diets can help public health practitioner’s target their efforts and inform public health policy. Given that these data show that stores provide the majority of foods for young children, parents may be the best intervention point. Educating parents on the dietary needs of their children and giving them the tools that they need to shop for and prepare healthy foods may result in long-term health benefits. Research is needed to look at how the number of meals and the source of foods influence nutritional intake and overall diet quality among young children. 

## Figures and Tables

**Table 1:** Selected demographic characteristics of US children aged 1 to 4 years ^1^, National Health and Nutrition Examination Surveys, 2009–2014 (*n* = 2640).

Characteristic	*n* ^2^	% ^3^ (95% Confidence Interval)
Total	2640	
Age		
1 to 2	1515	50.6 (47.8–53.3)
3 to 4	1125	49.4 (46.7–52.2)
Sex		
Male	1330	51.0 (48.2–53.9)
Female	1310	49.0 (46.1–51.8)
Race/Hispanic origin ^4^		
Non-Hispanic white	735	51.2 (45.4–57.1)
Non-Hispanic black	609	14.0 (11.1–17.0)
Hispanic	947	25.2 (20.0–30.3)
Ratio of family income to poverty threshold		
>1.85	858	46.7 (42.2–51.1)
≤1.85	1656	53.3 (48.9–57.8)
Day of intake		
Weekday (Mon–Fri)	1533	71.1 (68.9–73.3)
Weekend (Sat–Sun)	1107	28.9 (26.7–31.1)

^1^ Age at the time of exam in the Medical Examination Center (MEC). ^2^ Total population N = 2640; poverty variable missing data for 126 participants. ^3^ Data are weighted to account for the survey sampling design. ^4^ Race/Hispanic origin sub-analyses are limited to participants who report being either non-Hispanic white, non-Hispanic black, and Hispanic.

**Table 2:** Source ^1^ of all foods and drinks ^2^ reportedly consumed in a 24-h recall by children 1 to 4 years of age, National Health and Nutrition Examination Surveys, 2009–2014, *n* = 2640.

	Child Received Any Food from Child Care				Child Received No Food from Childcare			
	Total Population (*n* 273)	1- to 2-year-olds (*n* 94)	3- to 4-year-olds (*n* 179)	*P*-value (comparison of 1- to 2- and 3- to 4-year-olds)	Total Population (*n* 2367)	1- to 2-year-olds (*n* 1421)	3- to 4-year-olds (*n* 946)	*P*-value (comparison of 1- to 2- and 3- to 4-year-olds)
	% ^3^ (95% Confidence interval)	% (95% Confidence interval)	% (95% Confidence interval)		% ^3^ (95% Confidence interval)	% (95% Confidence interval)	% (95% Confidence interval)	
Store-bought	53.2 (48.7–57.7)	48.6 (43.6–53.6)	55.2 (49.9–53.6)	0.035	84.9 (83.6–86.1)	86.7 (85.2–88.2)	82.8 (80.5–85.0)	0.007
Restaurant	6.7 (4.3–9.1)	6.2 (2.3–10.1)	6.9 (4.4–9.3)	0.7236	8.9 (7.7–10.0)	7.4 (6.1–8.8)	10.5 (8.7–12.2)	0.0085
Childcare	36.2 (32.1–40.2)	41.8 (36.0–47.5)	33.7 (29.4–37.9)	0.0084	−	−	−	−
Other Source	3.8 (2.2–5.5)	3.3 (1.0–5.7)	4.1 (2.1–6.0)	<0.0001	5.5 (4.8–6.1)	4.5 (3.8–5.2)	6.5 (5.3–7.7)	0.0032

^1^ Store-bought includes grocery/supermarket, convenience store, and store (no additional info); Restaurants include restaurant, bar, sports, recreation, or entertainment facility, and street vendor or vending truck; Childcare includes cafeterias, child/adult care center, child/adult home care; Other source includes assistance programs, food grown or caught by you or someone you know, and any other listed source. ^2^ Tap water was excluded from this sub-analysis. ^3^ Percent of all food and drink items from each source was calculated using the ratio of the means and is based on one 24-h dietary recall; logistic regression was used to assess group differences.

## References

[1] Birch L.L., Fisher J.O. (1998). Development of eating behaviors among children and adolescents. Pediatrics.

[2] Savage J.S., Fisher J.O., Birch L.L. (2007). Parental influence on eating behavior: Conception to adolescence. J. Law Med. Ethics.

[3] Walker S.P., Wachs T.D., Grantham-McGregor S., Black M.M., Nelson C.A., Huffman S.L., Baker-Henningham H., Chang S.M., Hamadani J.D., Lozoff B. (2011). Inequality in early childhood: Risk and protective factors for early child development. Lancet.

[4] Black M.M., Quigg A.M., Hurley K.M., Pepper M.R. (2011). Iron deficiency and iron-deficiency anemia in the first two years of life: Strategies to prevent loss of developmental potential. Nutr. Rev..

[5] Baidal J.A.W., Locks L.M., Cheng E.R., Blake-Lamb T.L., Perkins M.E., Taveras E.M. (2016). Risk Factors for Childhood Obesity in the First 1000 Days: A Systematic Review. Am. J. Prev. Med..

[6] Pan L., Li R., Park S., Galuska D.A., Sherry B., Freedman D.S. (2014). A longitudinal analysis of sugar-sweetened beverage intake in infancy and obesity at 6 years. Pediatrics.

[7] US Department of Health and Human Services, US Department of Agriculture (2015). 2015–2020 Dietary Guidelines for Americans.

[8] Hagan J.F., Shaw J.S., Duncan P.M., American Academy of Pediatrics (2008). Promoting Healthy Nutrition. Bright Futures: Guidelines for Health Supervision of Infants, Children, and Adolescents.

[9] Popkin B.M., Duffey K.J. (2010). Does hunger and satiety drive eating anymore? Increasing eating occasions and decreasing time between eating occasions in the United States. Am. J. Clin. Nutr..

[10] US Department of Education, National Center for Education Statistics, National Household Education Surveys Program (NHES) Early Childhood Program Participation (ECPP) Survey; 2012. https://nces.ed.gov/pubs2013/2013029rev.pdf.

[11] National Health and Nutrition Examination Survey, 2009–2014 Continuous NHANES Data, Questionnaires and Related Documentation. https://wwwn.cdc.gov/nchs/nhanes/.

[12] Centers for Disease Control and Prevention (2009–2014). NHANES Response Rates and Population Totals. https://wwwn.cdc.gov/nchs/nhanes/responserates.aspx.

[13] Food and Nutrition Service (2016). WIC Income Eligibility Requirements.

[14] (2010). National Health and Nutrition Examination Survey Dietary Interviewer’s Procedure Manual.

[15] (2012). National Health and Nutrition Examination Survey Dietary Interviewer’s Procedure Manual.

[16] (2014). National Health and Nutrition Examination Survey Dietary Interviewer’s Procedure Manual.

[17] Murakami K., Livingstone M.B. (2016). Meal and snack frequency in relation to diet quality in US children and adolescents: The National Health and Nutrition Examination Survey 2003–2012. Public Health Nutr..

[18] National Health and Nutrition Examination Surveys Dietary Web Tutorials: Estimate Ratios. https://www.cdc.gov/nchs/tutorials/dietary/objectives.htm.

[19] Johnson C.L., Paulose-Ram R., Ogden C.L., Carroll M.D., Kruszan-Moran D., Dohrmann S.M., Curtin L.R. (2013). National health and nutrition examination survey: Analytic guidelines, 1999–2010. Vital Health Stat. Ser. 2 Data Eval. Methods Res..

[20] Institute of Medicine (US) Committee on Scientific Evaluation of WIC Nutrition Risk Criteria (1996). WIC Nutrition Risk Criteria: A Scientific Assessment.

[21] US Department of Agriculture, Food and Nutrition Services Child and Adult Care Food Program (CACFP) Website. https://www.fns.usda.gov/cacfp/child-and-adult-care-food-program.

[22] Drewnowski A., Rehm C.D. (2013). Energy intakes of US children and adults by food purchase location and by specific food source. Nutr. J..

[23] Holmes A.S., Estabrooks P.A., Davis G.C., Serrano E.L. (2012). Effect of a grocery store intervention on sales of nutritious foods to youth and their families. J. Acad. Nutr. Diet..

[24] Milliron B.J., Woolf K., Appelhans B.M. (2012). A point-of-purchase intervention featuring in-person supermarket education affects healthful food purchases. J. Nutr. Educ. Behav..

[25] Zoumas-Morse C., Rock C.L., Sobo E.J., Neuhouser M.L. (2001). Children’s patterns of macronutrient intake and associations with restaurant and home eating. J. Am. Diet. Assoc..

